# Retinal ganglion cell desensitization is mitigated by varying parameter constant excitation pulse trains

**DOI:** 10.3389/fncel.2022.897146

**Published:** 2022-08-12

**Authors:** Wennan Li, Dorsa Haji Ghaffari, Rohit Misra, James D. Weiland

**Affiliations:** ^1^Department of Biomedical Engineering, University of Michigan, Ann Arbor, MI, United States; ^2^Department of Ophthalmology and Visual Sciences, University of Michigan, Ann Arbor, MI, United States; ^3^Biointerfaces Institute, University of Michigan, Ann Arbor, MI, United States

**Keywords:** retinal ganglion cell (RGC), desensitization, electrical stimulation, retinal prosthesis, picrotoxin

## Abstract

Retinal prostheses partially restore vision in patients blinded by retinitis pigmentosa (RP) and age-related macular degeneration (AMD). One issue that limits the effectiveness of retinal stimulation is the desensitization of the retina response to repeated pulses. Rapid fading of percepts is reported in clinical studies. We studied the retinal output evoked by fixed pulse trains vs. pulse trains that have variable parameters pulse-to-pulse. We used the current clamp to record RGC spiking in the isolated mouse retina. Trains of biphasic current pulses at different frequencies and amplitudes were applied. The main results we report are: (1) RGC desensitization was induced by increasing stimulus frequency, but was unrelated to stimulus amplitude. Desensitization persisted when the 20 Hz stimulation pulses were applied to the retinal ganglion cells at 65 μA, 85 μA, and 105 μA. Subsequent pulses in the train evoked fewer spikes. There was no obvious desensitization when 2 Hz stimulation pulse trains were applied. (2) Blocking inhibitory GABA_A_ receptor increased spontaneous activity but did not reduce desensitization. (3) Pulse trains with constant charge or excitation (based on strength-duration curves) but varying pulse width, amplitude, and shape increased the number of evoked spikes/pulse throughout the pulse train. This suggests that retinal desensitization can be partially overcome by introducing variability into each pulse.

## Introduction

Retinal prostheses are implantable electronic devices designed to restore useful vision in blind patients through electrical stimulation of the remaining inner retinal neurons (Bloch et al., 2019). From the first report of the implantation of a photovoltaic array in the suprachoroidal space of a blind volunteer (Tassicker, 1956), steady progress has yielded several devices with market approval [Argus II (Second Sight, USA; Humayun et al., 2003), Alpha IMS (Retina Implant AG, Germany; Zrenner et al., 2011), IRIS II (Pixium, France), and Bionic Eye System (Bionic Vision Technologies, Australia; Ayton et al., 2014) or in clinical trials, including Prima (Pixium; Palanker et al., 2020)]. Retinal implants allow patients to recognize shapes, perceive the contrast between light and dark objects, and identify large letters (Zrenner et al., 2011; Humayun et al., 2012; Stingl et al., 2013; Ayton et al., 2014; Bloch et al., 2019). Although retinal prostheses improve the overall quality of life for patients (Humayun et al., 2012), the implants are not a replacement for normal vision due to the limitations (reproducibility of phosphenes, limited stimulation frequencies, low spatial resolution, etc.).

Several clinical studies have revealed that phosphenes become less bright with continuous stimulation in retinal prosthesis patients (Zrenner et al., 2011; Fornos et al., 2012; Stingl et al., 2017). In Argus II patients, the phosphene faded over several seconds, and some faded in less than 1 s (Fornos et al., 2012; Stronks et al., 2013; Stronks and Dagnelie, 2014; Weiland and Humayun, 2014). It has been reported that using relatively low stimulation rates can prevent fading, but this may result in “blinking” percepts, as noted in the Alpha-IMS patients when 5 Hz stimulation was used (Zrenner et al., 2011; Stingl et al., 2013, 2015). This significantly detracts from the usefulness of these systems.

The reduction of retinal ganglion cell (RGC) responses to repetitive electrical stimulation is referred to as desensitization (Jensen and Rizzo, 2007; Freeman and Fried, 2011; Im and Fried, 2016). This impedes high temporal resolution (Jensen and Rizzo, 2007; Fornos et al., 2012; Stingl et al., 2015; Höfling et al., 2020) and prevents the creation of continuous percepts. Studies have shown that the sensitivity of rabbit retinal ganglion cells to electrical stimulation progressively decreased with repeated stimulation at certain stimulation frequencies and this desensitization persisted in the presence of amacrine cell inhibition (Freeman and Fried, 2011). Other studies have also shown that the network mediated responses of ganglion cells to stimulation at 20 Hz would typically induce strong fading in mice (Sekhar et al., 2016) and rat retina (Sekirnjak et al., 2006). Although these findings suggest decreasing pulse frequency can eliminate perceptual fading, reducing frequency will lead to flickering and less effective artificial vision. Here, we replicate the findings showing desensitization and demonstrate a possible solution, which involves varying pulse parameters such as pulse width and amplitude, while maintaining the overall level of excitation provided by each pulse constant. This approach results in a more robust RGC response at frequencies that desensitize RGC output when constant pulse parameters are used.

## Materials and Methods

### Preparation of the retina

All procedures were approved by the Institutional Animal Care and Use Committee at the University of Michigan. C57BL/6 mice aged 6–10 weeks were used in this study. Mice were anesthetized with an intraperitoneal (IP) injection of ketamine (100 mg/kg)/xylazine (10 mg/kg). Cornea, lens, and vitreous body were removed, and the retina was isolated from the pigment epithelium and cut into four pieces. Retina pieces were placed on filter article with ganglion cells layer up. The filter article with tissue was mounted in a chamber of an upright microscope (Olympus BX51WI) with 40× water immersion lenses. The chamber was continuously perfused with Ames solution (Sigma-Aldrich, St. Louis, MO USA; 4–6 ml/min) and equilibrated with 95% O_2_ 5% CO_2_ at 37°C.

### Electrophysiological recordings

Current-clamp recording was used in this study using HEKA EPC10 (Warner Instrument) and was performed by making a hole in the inner limiting membrane with the glass pipette filled with an internal solution. Positive pressure was applied to expose RGCs. Cells 15–20 μm in diameter were selected for recording. The electrode internal solution contained (in mM): K-gluconate111, NaCl 5, KCl4, EGTA 2, HEPES 10, Mg-ATP4, Na-GTP 0.3, Tris2-phosphocreatine 7; (mOsm = 275, pH = 7.3). Pipette resistance at the beginning of the recordings was 3–6 MΩ.

### Picrotoxin

Picrotoxin solution was prepared in normal Ames’ solution containing DMSO to a maximal concentration of 0.1%. After testing thresholds of the cells, Picrotoxin (100 μM) was applied in the perfusion system to the bath by switching a three-way stopcock and 4–6 min was allowed for the drug to wash in and take effect. This is similar to the concentration used in other studies with isolated retina (Freeman and Fried, 2011).

### Electrical stimulation

A single Pt-Ir disk electrode with a diameter of 75 μm was used for electrical stimulation, which was placed ~70 μm away and ~50 μm above the targeted retinal ganglion cell’s soma before performing the current-clamp patching (Cho et al., 2011). The ground electrode was placed behind the retina on the photoreceptor side. Stimulus pulses were generated using Multi-channel systems stimulus generator (STG 4008, Germany) in MC stimulus II software.

### Threshold measurement

Amplitude for current stimuli was initially set to 25 μA and was increased in 20 μA steps until each pulse evoked at least one spike. Dose-response curves were created by fitting a logistic equation to the spike probability values vs. current amplitudes (1). The threshold was defined as the current value (in the equation below) when *p* = 0.5 (Cho et al., 2016).


(1) $$p=\frac{a}{b+e^{-xc}}$$

Where *p* is the spike probability, *x* is the current amplitude, and *a*, *b* and *c* are constants.

### Fixed parameter pulse trains characterize desensitization

We used pulses of fixed amplitude and duration to characterize desensitization. Pulse shape was cathodic-first, biphasic, with pulse width and interphase gap all set to 0.5 ms. Fixed pulse amplitudes of 65, 85, and 105 μA were used since these amplitudes were typically suprathreshold. Twenty pulses were delivered at 2 Hz and 20 Hz.

### Varying parameter constant excitation (VPCE) pulse trains

We created four different sets of varying pulse trains, to compare fixed pulse trains. Pulse parameters, including pulse width, amplitude, polarity order, phase ratio (ratio of the anodic to cathodic phase duration), and interphase gap (IPG) were varied such that no two consecutive pulses were the same. We maintained constant excitation on each pulse in a train in two ways: (1) maintaining stimulus charge or (2) scaling charge according to a strength duration relationship (see details below). Pulse protocols are defined as follows: (1) Only pulse width is varied and charge per phase is kept constant. (2) Only pulse width is varied and charge per phase is calculated based on the strength-duration curve. (3) All parameters are varied and charge per phase is kept constant. (4) All parameters are varied and charge per phase is calculated based on the strength-duration curve. Because each cell can only be patched for a limited time, we tested VCPE trains with 105 μA, 0.5 ms biphasic, cathodic-first as the baseline. The pulse rate was maintained at 20 Hz. Using the baseline pulse and an RGC chronaxie of 0.33 ms (Sekirnjak et al., 2006; Chan et al., 2011), we calculated an asymptotic current (rheobase) of 83 μA, then used these values for rheobase and chronaxie to calculate pulse amplitude and duration, according to the relationship in equation (2).


(2) $$a=\frac{irh}{1-e^{\frac{-d}{\tau }}}$$

Where *irh* is the rheobase current, τ is the chronaxie, *d* is the pulse width, and *a* is pulse amplitude.

**Figure 1:**
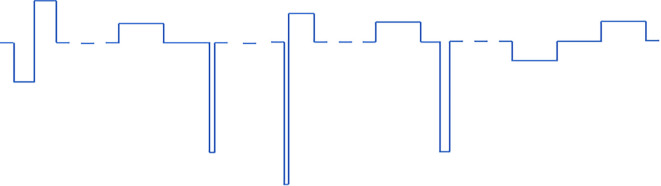
An example of one of the VPCE pulse trains (protocol 3), where constant charge/phase was maintained in each pulse but pulse parameters are varied. The sequence shown is repeated four times in a 20 pulse sequence.

In protocols 1 and 2 (pulse width varied) there are six different pulse type that get repeated periodically. In protocols 3 and 4 (all parameters varied) there are five different combinations of pulse parameters that get repeated periodically. All VPCE pulse trains were delivered at 20 Hz. An example pulse train from protocol 3 is shown in Figure 1. Corresponding pulse parameters are shown in Table 1. Pulse parameters for protocols 1, 2, and 4 are provided as Supplementary Tables.

**Table 1:** Pulse parameters for protocol 3.

**Pulse #**	**Phase 1 PW (ms)**	**Phase 1 amplitude (μA)**	**IPG (ms)**	**Phase 2 PW (ms)**	**Phase 2 amplitude (μA)**	**Charge density per phase (mC/cm^2^)**
1	0.5	−105	0	0.5	105	0.297
2	1	52.5	1	0.1	−525	0.297
3	0.06	−875	0	0.6	87.5	0.297
4	1	52.5	0.5	0.2	−262.5	0.297
5	1	−52.5	1	1	52.5	0.297

### Data analysis and statistics

Action potential peaks within 5 ms of the pulse onset were counted as directly evoked spikes, originating in RGCs, and peaks within 50 ms of pulse onset were considered indirectly evoked spikes, originating in the inner nuclear layer (Boinagrov et al., 2014). The raw data were processed by using the analysis software Fitmaster (HEKA) and was exported to MATLAB for further analysis. Outliers were identified using the Z score for Outlier Detection: Z score = (x-mean)/std. deviation. For studying the effect of VPCE pulse trains we used a linear mixed model (Harrison et al., 2018) with the total number of evoked spikes as the response variable, protocol number as a fixed effect variable and cell number (1–29) as a random effect variable. Intrinsic differences in RGC responses refer to RGC activation thresholds in response to electrical stimulation. This difference could be based on morphological and physiological variations between at least 20 types of RGCs in the retina (Fohlmeister and Miller, 1997; Masland, 2011). Different spontaneous firing rates and baseline membrane potentials of RGCs also contribute to variability in stimulation thresholds (Cho et al., 2016). This model was chosen to determine if the total spike number evoked by VPCE protocols is significantly different than the control pulse train considering the inherent variability in RGC responses. This analysis was done using the fitlme function in MATLAB and by incorporating the following equation:


(3) y∼p+(1|c)

Where *y* is the total number of spikes evoked by a VPCE pulse train, *p* is the protocol number (categorical variable) and *c* is the cell number.

We also used linear mixed models to evaluate the effect of amplitude and picrotoxin on the total number of evoked spikes with different stimulation frequencies. In this case, the total number of evoked spikes was the response variable, the variable under study (amplitude or picrotoxin) was the fixed effect variable and the cell number was the random effect variable. In all analyses, the response to the first three pulses (first 150 ms) was not included, since rapid desensitization occurs in that time window before RGC responses reach a steady state (Freeman and Fried, 2011).

## Results

### Effect of stimulation frequency and amplitude on RGC desensitization

**Figure 2:**
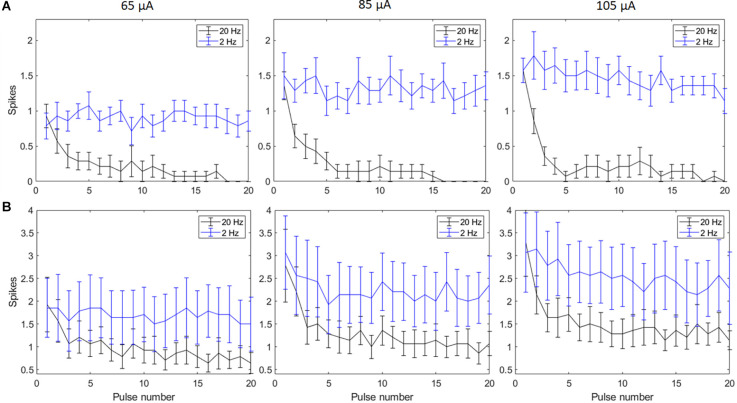
Spiking activity of RGCs in response to electrical stimulation (*n* = 14). The average number of RGC spikes counted in 5 ms **(A)** and 50 ms bins **(B)** in response to 20 pulses (0.5 ms/phase) delivered at three current amplitudes (65 μA, 85 μA, and 105 μA) at pulse rates of 2 Hz and 20 Hz. Error bars show the standard error of the mean (SEM).

Biphasic cathodic first pulses (0.5 ms/phase, 0.5 ms IPG) were applied at three current amplitudes (65 μA, 85 μA, and 105 μA) at pulse rates of 2 Hz and 20 Hz. Figures 2A,B show spike counts in 5 ms and 50 ms bins after the onset of each pulse (*n* = 14). For 20 Hz stimulation, the number of evoked spikes diminished with subsequent pulses relative to the first pulse at all stimulation amplitudes, while the number of evoked spikes was consistent for all pulses in the 2 Hz train. Result of the linear mixed model for spike count in 5 ms bins (Figure 2A) shows a significant increase in evoked spikes with 85 and 105 μA current amplitudes compared to 65 μA at 2 Hz (*p* < 0.01**, *p* < 0.001***). There was no significant change in the total number of evoked spikes with different amplitudes at 20 Hz. For spike count in 50 ms bins (Figure 2B) there was a significant increase in evoked spikes with 85 and 105 μA current amplitudes compared to 65 μA at 2 Hz (*p* < 0.01**, *p* < 0.001***). At 20 Hz, there was a significant increase in evoked spikes with 105 μA compared to 65 μA (*p* < 0.001***).

### Effect of PTX on the RGC activity

**Figure 3:**
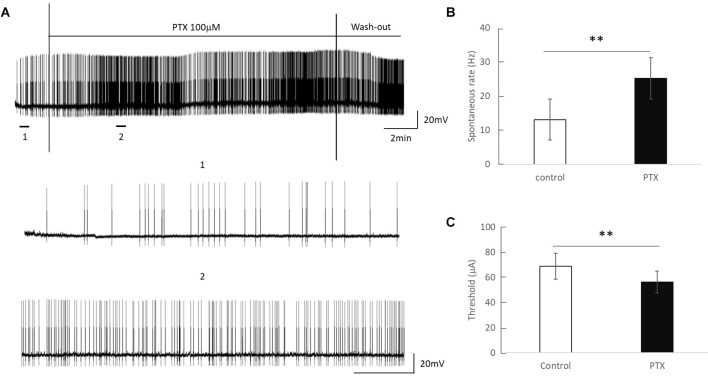
The effect of PTX (100 μM) on the spontaneous spike rate of RGCs. **(A)** Spontaneous spikes of an RGC before and after application of PTX. One and two show zoomed-in traces for before and after application of PTX respectively. **(B,C)** PTX significantly increased spontaneous spike rate (**p < 0.01) and decreased the threshold (**p < 0.01) compared to control.

Amacrine cells release inhibitory neuron transmitters including GABA. Prior work has shown that amacrine cells may inhibit RGC spiking during repetitive pulsing (Fried et al., 2006). Thus we examined the RGC excitability in the presence of picrotoxin (100 μM), the antagonist of the GABA_A_ receptor to partially reduce this inhibition. Figure 3 shows representative spikes trains before and after PTX was applied. RGC spontaneous spike rate increased after administration of PTX (Before PTX: 13.16 ± 3.85 spikes/s; After: 25.27 ± 4.65 spikes/s, *n* = 24, *p* < 0.01**). RGC thresholds decreased after administration of PTX (Before PTX: 68.72 ± 10.33 μA; after PTX: 56.33 ± 8.68 μA, *n* = 29 *p* < 0.01**). These implied that blocking amacrine cells’ GABA_A_ receptor increased the excitability of RGCs as reflected by a decrease in activation thresholds.

**Figure 4:**
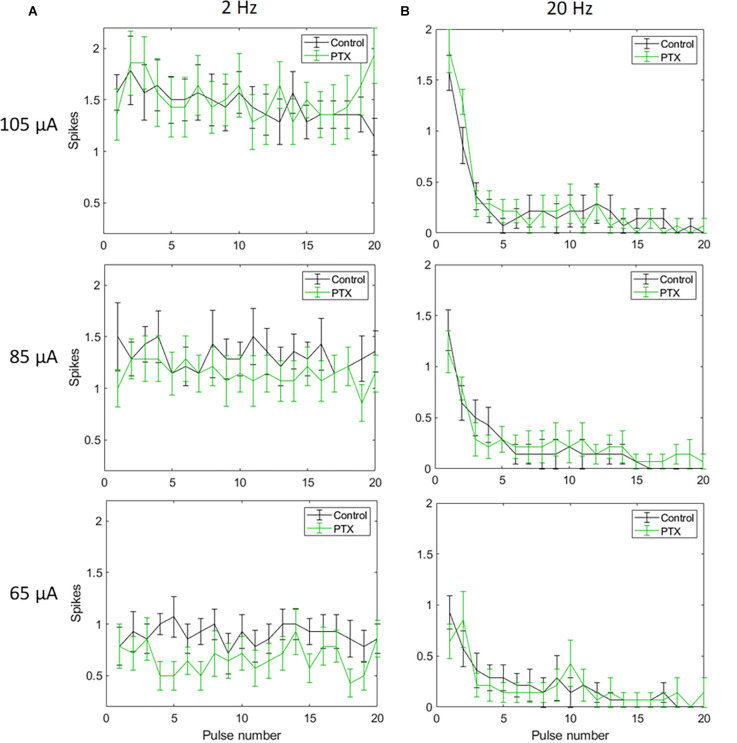
The effect of PTX on RGC spiking (*n* = 14) at different current amplitudes for stimulation delivered at 2 Hz **(A)** and 20 Hz **(B)**. The average numbers of RGC spikes in response to 20 pulses delivered at three current amplitudes (65 μA, 85 μA, and 105 μA) are shown in each figure. Evoked spikes are counted in 5 ms bins after the onset of each pulse. Error bars show the standard error of the mean (SEM).

**Figure 5:**
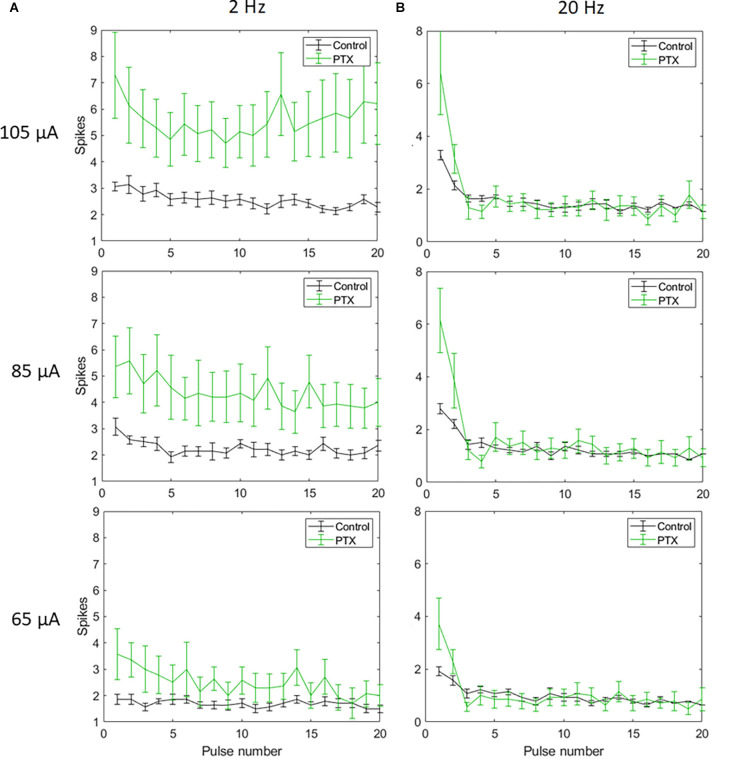
The effect of PTX on RGC spiking (*n* = 14) at different current amplitudes for stimulation delivered at 2 Hz **(A)** and 20 Hz **(B)**. The average numbers of RGC spikes in response to 20 pulses delivered at three current amplitudes (65 μA, 85 μA, and 105 μA) are shown in each figure. Evoked spikes are counted in 50 ms bins after the onset of each pulse. Error bars show the standard error of the mean (SEM).

With spike count in 5 ms bins (Figures 4A,B; *n* = 14) no significant change in total spike count was observed with PTX addition. With spike count in 50 ms bins for 2 Hz stimulation (Figure 5A), adding PTX resulted in a significant increase in total spike counts only for 85 and 105 μA stimulation amplitudes. For 20 Hz stimulation (Figure 5B) no significant change in spike count was observed with PTX. These results indicate that PTX did not reduce desensitization. This is consistent with results from Freeman 2011, who found that desensitization was not eliminated after blocking (Freeman and Fried, 2011).

### Effect of VPCE pulse trains on RGC desensitization

**Figure 6:**
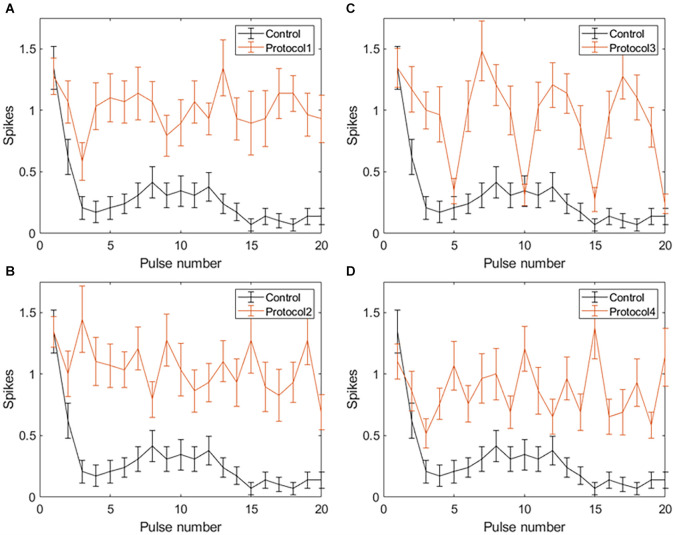
Spiking activity of RGCs in response to VPCE pulse trains and control pulse train at 20 Hz. Number of evoked spikes counted in 5 ms bins after the onset of each pulse is plotted against the pulse number (*n* = 29). The pulse sequences in Table 1, Supplementary Tables S1, S2, and S3 are repeated to create a train of 20 pulses. Spiking activity in response to **(A)** Protocol 1: pulse width varies and charge per phase is kept constant. **(B)** Protocol 2: pulse width varies and charge per phase is calculated based on strength-duration curve. **(C)** Protocol 3: all pulse parameters vary and the charge per phase is kept constant. **(D)** Protocol 4: all pulse parameters vary and charge per phase is calculated based on strength-duration curve.

**Figure 7:**
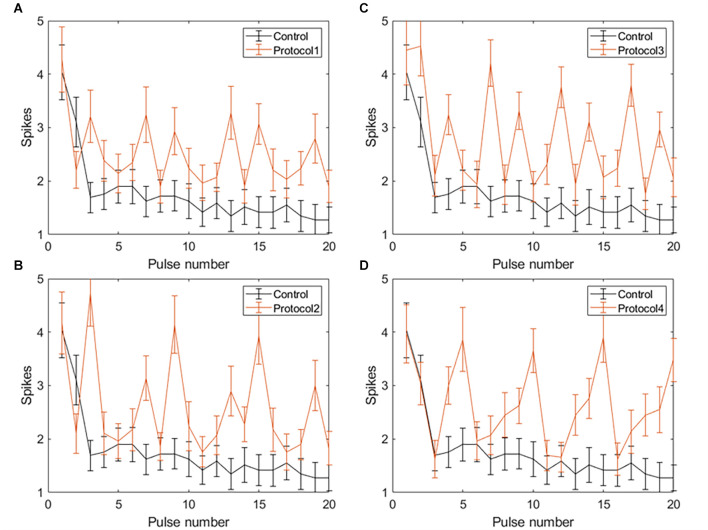
Spiking activity of RGCs in response to VPCE pulse trains and control pulse train at 20 Hz. The number of evoked spikes counted in 50 ms bins after the onset of each pulse is plotted against the pulse number (*n* = 29). The pulse sequences in Table 1, Supplementary Tables S1, S2, and S3 are repeated to create a train of 20 pulses. Spiking activity in response to **(A)** Protocol 1: pulse width varies and charge per phase is kept constant. **(B)** Protocol 2: pulse width varies and charge per phase is calculated based on strength-duration curve. **(C)** Protocol 3: all pulse parameters vary and the charge per phase is kept constant. **(D)** Protocol 4: all pulse parameters vary and charge per phase is calculated based on strength-duration curve.

Applying VPCE pulse trains increased the RGC response rate during continuous pulsing at 20 Hz. RGC spike counts increased for all four VPCE protocols, but in a different way for each protocol (*n* = 29). Figures 6 and 7 show the effect of different protocols on spike numbers counted in 5 ms and 50 ms bins after the pulse onset with 20 consecutive pulses. The total number of evoked spikes was analyzed using a linear mixed model. The protocol number was considered a fixed effect variable and the cell number (1–29) was considered a random effect variable. Results from this analysis show that all VPCE pulse protocols evoked a significantly higher number of spikes compared to the control pulse train. The rate of increase in total evoked spike counts in 5 ms bins (Figure 6) was 362%, 358%, 307%, and 299% for protocols 1, 2, 3, and 4 respectively. The rate of increase in total evoked spike counts in 50 ms bins (Figure 7) was 53.99%, 56.21%, 69.54%, and 67.97% for protocols 1, 2, 3, and 4 respectively. Observations for each protocol are given below.

#### Spike count in 5 ms bins after pulse onset

Average total spikes ± SEM for the control pulse train = 3.75 ± 1.21.

**Protocol 1**: Pulse width is varied; charge is kept constant (Figure 6A). No patterns were noted between spike numbers evoked by consecutive pulses (average total spikes ± SEM = 17.38 ± 2.51).

**Protocol 2**: Pulse width is varied; charge is calculated based on the SD curve (Figure 6B). No patterns were noted between spike numbers evoked by consecutive pulses (average total spikes ± SEM = 17.24 ± 2.47).

**Protocol 3**: All pulse parameters are varied; charge is kept constant (Figure 6C). Pulses #5, 10, 15, and 20 generate a significantly lower number of spikes compared to other pulses. These pulses are symmetric cathodic-first with 1 ms pulse width and 1 ms IPG (Table 1; average total spikes ± SEM = 15.31 ± 2.58).

**Protocol 4**: All pulse parameters are varied; charge is calculated based on the SD curve (Figure 6D). No patterns were noted between spike numbers evoked by consecutive pulses (average total spikes ± SEM = 15 ± 2.19).

#### Spike count in 50 ms bins after pulse onset

Average total spikes ± SEM for the control pulse train = 26.38 ± 6.56.

**Protocol 1**: Pulse width is varied; charge is kept constant (Figure 7A). No patterns were noted between spike numbers evoked by consecutive pulses (average total spikes ± SEM = 40.62 ± 6.35).

**Protocol 2**: Pulse width is varied; charge is calculated based on SD curve (Figure 7B). Largest responses (more spikes/pulse) were generated by a 1 ms, symmetric cathodic-first pulse (pulse #3, 9, 15 see Supplementary Table S2). These pulses were preceded by pulses of lower charge (average total spikes ± SEM = 41.2 ± 5.89).

**Protocol 3**: All pulse parameters are varied; the charge is kept constant (Figure 7C). The largest responses were generated by pulse #2 (Table 1), asymmetric anodic-first with a short, high amplitude cathodic 2nd phase. Our prior work (Chang et al., 2019; Haji Ghaffari et al., 2020) has shown that this type of pulse has a lower threshold compared to standard symmetric cathodic-first pulses (average total spikes ± SEM = 44.72 ± 6.6).

**Protocol 4**: All pulse parameters are varied; charge is calculated based on the SD curve (Figure 7D). A significantly larger response was generated by pulse #5, symmetric cathodic-first with 1 ms pulse width, with the largest charge (Supplementary Table S3). Here, asymmetric anodic-first pulses are not as effective as in protocol 3, but due to the scaling of charge by the SD relationship, the asymmetric anodic-first pulses in protocol 4 had less charge than those in protocol 3 (average total spikes ± SEM = 44.31 ± 5.26; refer to Supplementary Figure S2 for PSTH of all protocols).

## Discussion

Prosthetic vision has helped improve light perception, motion detection, and performance in visually guided tasks for users blinded by retinal degenerative diseases (Weiland et al., 2005; Zrenner, 2013; Edwards et al., 2018). However, limitations such as the low resolution of stimulation, and fading of percepts make it challenging to perceive a continuous, high quality image (Weiland and Humayun, 2014; Erickson-Davis and Korzybska, 2021). Retinal implant users are instructed to perform head movements to counteract fading of percepts that is due to neural adaptation in the retina and visual cortex (Hsieh and Colas, 2012). On the level of the retina, neural adaptation is thought to be caused by intrinsic desensitization of bipolar cells, activation of presynaptic inhibitory networks, and desensitization of sodium channels in the RGC membrane (Freeman and Fried, 2011; Soto-Breceda et al., 2018; Walston et al., 2018).

We studied the retinal output evoked by fixed pulse trains vs. pulse trains that have variable parameters pulse-to-pulse, to test our hypothesis that VPCE trains will mitigate desensitization. Our main findings were as follows: (1) RGC desensitization was induced by increasing stimulus frequency but was unrelated to stimulus amplitude. There was no obvious desensitization when 2 Hz stimulation pulses were applied. (2) Blocking GABA_A_ receptor did not abolish the desensitization. (3) Applying a pulse train where pulse parameters varied (VPCE) resulted in less desensitization. The number of evoked spikes by electrical stimulation did not continuously decrease after the first pulse, but the spike/pulse rate was not stable as it was at 2 Hz.

A number of studies have demonstrated desensitization in RGCs. Freeman and Fried (2011) showed two time scales for desensitization in RGCs, rapid and slow, and that desensitization is independent of amacrine cell inhibition. Fornos et al. (2012) showed that Argus II retinal prosthesis users report rapid fading of percepts and changing of percepts into dimmer and poorly localized percepts, which interrupts the perception of a meaningful image. Weitz et al. (2014) showed an increase in Argus II perception thresholds over time which indicated that the retina was getting desensitized. Several studies showed that inhibitory GABAergic and glycinergic feedback from amacrine cells truncates release from the bipolar cell terminals to generate phasic output signals (Awatramani and Slaughter, 2000). PTX may also combine GABAa receptors on other retinal cells (e.g., bipolar cells) and decrease vesicle release to ganglion cells. And indirect stimulation of other retinal neurons causes feedback between them and RGCs, which modulates RGCs desensitization. However, inhibitory feedback is not the sole cause of desensitization, since Freeman and Fried (2011) found that desensitization remained after blocking amacrine cell input to bipolar cells. These studies demonstrated desensitization and possible mechanisms underlying it but did not propose stimulation strategies to minimize this effect.

Prior studies have assessed varying pulses to reduce desensitization. Our prior work on this topic looked over a longer time period (compared to the 20 pulses applied in the current study). During 1 h of epiretinal stimulation in the rat model, the evoked response strength (recorded at the superior colliculus) was measured every 5 min while applying either constant pulse trains or “time-varying” trains (Davuluri and Weiland, 2014). We noted that the evoked response strength decreased less during 1 h of stimulation when “time-varying” trains were used. Another study also showed that randomizing the inter-pulse interval can lower the response decay rate at 50 and 200 Hz but not with 10 Hz stimulation (Soto-Breceda et al., 2018). Chenais et al. (2021) demonstrated that a naturalistic stimulation strategy where electrical pulses are modulated spatiotemporally could mitigate the desensitization of RGCs in mouse retina. In this study, we varied pulse parameters so that no two consecutive pulses are the same, but we kept pulse frequency constant (20 Hz). Consistent with the prior work, our study found less sensitization when with time-varying pulse trains vs. constant pulse trains.

Electrical stimulation activates the retina in two ways: Direct stimulation which depolarizes RGCs and evokes action potentials (spikes), and network stimulation which depolarizes retinal cells presynaptic to RGCs, and evokes RGC spikes through synapses to RGCs. Prior work (Fried et al., 2006; Sekirnjak et al., 2006; Ahuja et al., 2008) has shown that network responses attenuate rapidly at about 10 pps and are completely absent at 20 pps. Inhibitory feedback from amacrine cells is one suspected mechanism (Fried et al., 2006), although attenuation of bipolar cell responses has been shown recently (Walston et al., 2018). Network responses are typified by a burst of RGC spikes after a single electrical pulse. In contrast, direct stimulation typically evokes a single spike and that can be maintained at a frequency up to 100 Hz (Sekirnjak et al., 2006). A study of RGC responses at 2 and 16 pps found that the number of RGC spikes are decreased after the first pulse and blocking amacrine cells did not show a significant difference between the control and the blocker group at 2 and 16 Hz (Freeman and Fried, 2011), which matches our findings using 2 Hz and 20 Hz frequencies. In both the Freeman and our study, higher frequency pulse trains did evoke single spikes, which likely represent direct responses. We observed that an amplitude of 105 μA caused a significant increase in the number of indirectly evoked spikes compared to a 65 μA amplitude (Figure 2B). This is consistent with previous studies showing that synaptically evoked spikes (long latency) have higher current thresholds than directly evoked spikes (short latency) at a certain pulse width (Jensen et al., 2005). This suggests that higher current amplitudes are effective in increasing indirect RGC activation, but desensitization still persists with all amplitudes.

Applying VPCE pulse trains with 20 Hz frequency showed a significant increase in the total number of evoked spikes with all four protocols we tested. However, with all VPCE protocols, we observed variations in the evoked spike numbers with each consecutive pulse. RGCs show persistent desensitization in response to the control 20 Hz stimulation and this is evident in the persistently lowered number of spikes after the initial three pulses. This is in agreement with a previous research study showing a rapid desensitization mechanism for RGC spiking as well as a slow desensitization mechanism (Freeman and Fried, 2011). A similar pattern has been shown for the fading of percepts in retinal prosthesis users (Fornos et al., 2012), suggesting that the decline in RGC firing may be responsible for fading of percepts. Protocols 1, 2, and 4 provided a more stable spike count over time for directly evoked responses (Figure 6), which can be beneficial in providing a more stable image perception over time. The largest increases in the average spike numbers for indirectly evoked responses (Figure 7) were in response to longer pulse widths (1 ms), which may preferentially engage network stimulation. An exception to the general trend occurred with protocol 3, where pulse #5 (symmetric cathodic-first with 1 ms pulse width and 1 ms IPG) generates a significantly lower number of directly evoked spikes compared to other pulses (Figure 6C). This may be due to the constant charge approach used in protocol 3 and the known behavior that threshold charge increases with pulse width (Geddes and Bourland, 1985). In addition, longer pulses are known to preferentially evoke network activation (Fried et al., 2006). The same effect with the 1 ms pulse is observed with protocol 1, but not as pronounced as in protocol 3. It is unclear why these 1 ms pulses behave differently in protocols 1 and 3, but we speculate that the preceding pulse has some effect. Future studies of desensitization should consider the order of delivered pulses. For example, a long anodic pulse (1st phase) may not evoke a spike but can increase the probability of a cell spiking if the cathodic 2nd phase is delivered subsequently (Chang et al., 2019; Haji Ghaffari et al., 2020). In this study, we focused on temporal aspects of RGC desensitization. Spatial desensitization of RGCs can occur with interpulse distances smaller than 800 μm (Jalligampala et al., 2016), and should be taken into account during multi-electrode stimulation. We did not study the effect of VPCE on different classes of RGCs (*e.g.*, ON vs. OFF). Investigating VPCE on different morphological and physiological categories of RGCs will be an interesting future direction (Sanes and Masland, 2015). With photoreceptor degeneration, morphological changes can occur in the inner retina layers (Strettoi et al., 2003). It is shown that the spontaneous activity of RGCs increases with retinal degeneration (Stasheff, 2008). In addition, average RGC membrane potential is shown to be lower and activation thresholds in response to electrical stimulation are generally higher with retinal degeneration (Cho et al., 2016). Higher thresholds may affect how VPCE evokes RGC responses, for example, higher current amplitudes may be needed to stimulate RGCs. Results of this study can have implications for designing more effective stimulation protocols for retinal prostheses but will require more sophisticated stimulator chips to implement this technical solution.

## Data Availability Statement

The raw data supporting the conclusions of this article will be made available by the authors, without undue reservation.

## Ethics Statement

The animal study was reviewed and approved by Institutional Animal Care and Use Committee at the University of Michigan.

## Author Contributions

WL, DH, and JW designed the study and wrote the manuscript. WL performed the *in vitro* experiments. WL and DH analyzed the data. RM helped with design of pulse trains. All authors contributed to the article and approved the submitted version.

## Funding

This research was supported by University of Michigan and National Eye Institute (#EY022931 and # EY013934).
