# Supplementary material for: Retinal ganglion cell desensitization is mitigated by varying parameter constant excitation pulse trains
Source: Front Cell Neurosci. 2022 Aug 12;16:897146. doi: 10.3389/fncel.2022.897146 (PMC9407683; doi:10.3389/fncel.2022.897146)
Supplement: Supplementary file 1 [file Data_Sheet_1.PDF]

Table S1. Pulse parameters for protocol 1. Pulses are repeated consecutively to create a train of 20 pulses.

Pulse #	Phase 1 PW (ms)	Phase 1 amplitude (μA)	IPG (ms)	Phase 2 PW (ms)	Phase 2 amplitude (μA)	Charge density per phase (mC/cm^2)
1	0.5	-105	0	0.5	105	0.297
2	0.1	-525	0	0.1	525	0.297
3	1	-52.5	0	1	52.5	0.297
4	0.6	-87.5	0	0.6	87.5	0.297
5	0.06	-875	0	0.06	875	0.297
6	0.2	-262.5	0	0.2	262.5	0.297

Table S2. Pulse parameters for protocol 2. Pulses are repeated consecutively to create a train of 20 pulses.

Pulse #	Phase 1 PW(ms)	Phase 1 amplitude (μA)	IPG (ms)	Phase 2 PW (ms)	Phase 2 amplitude (μA)	Charge density per phase (mC/cm^2)
1	0.5	-105	0	0.5	105	0.297
2	0.1	-315	0	0.1	315	0.178
3	1	-87.2	0	1	87.2	0.493
4	0.6	-96.2	0	0.6	96.2	0.327
5	0.06	-490	0	0.06	490	0.166
6	0.2	-183.5	0	0.2	183.5	0.208

Table S3. Pulse parameters for protocol 4. Pulses are repeated consecutively to create a train of 20 pulses.

Pulse #	Phase 1 PW (ms)	Phase 1 amplitude (μA)	IPG (ms)	Phase 2 PW (ms)	Phase 2 amplitude (μA)	Charge density per phase (mC/cm^2)
1	0.5	-105	0	0.5	105	0.297
2	1	31.5	1	0.1	-315	0.178
3	0.06	-490	0	0.6	49	0.166
4	1	36.7	0.5	0.2	-183.5	0.208
5	1	-87.2	1	1	87.2	0.493

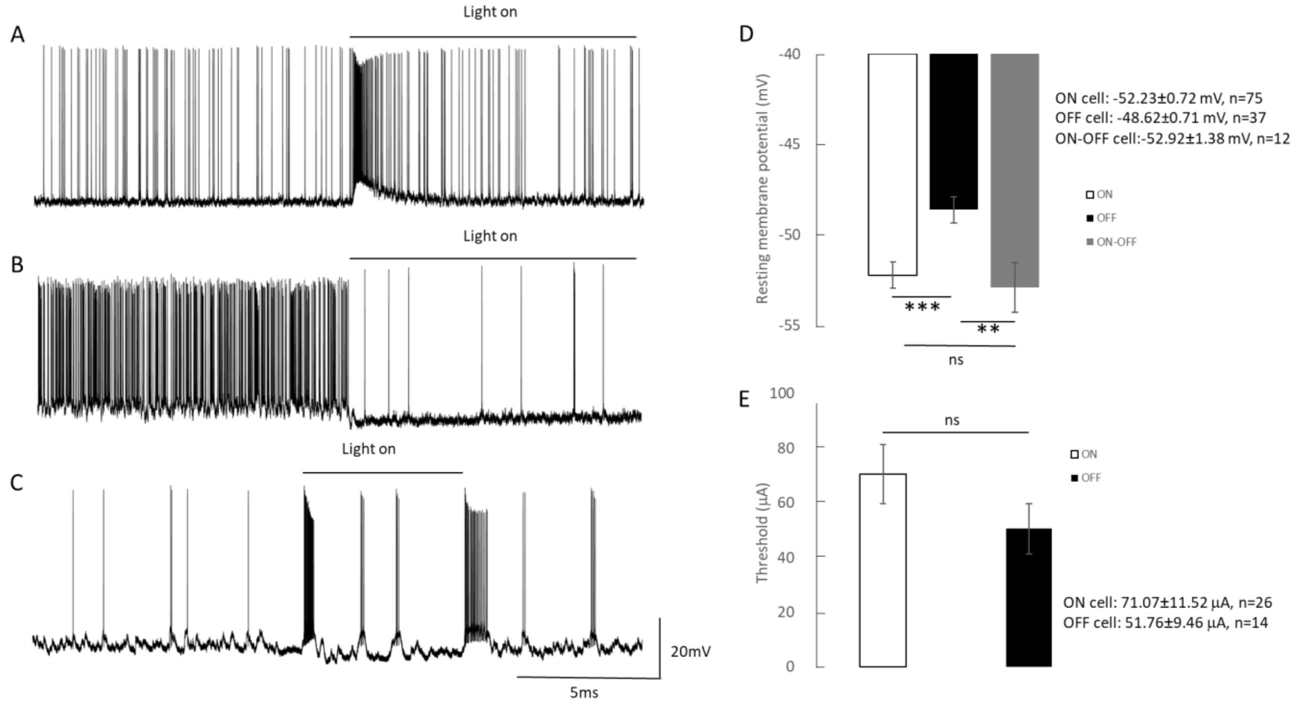

Figure S1. Spontaneous spiking for different RGC types. A-C) whole-cell current-clamp recordings in response to 6-10 s of light stimulus for ON, OFF, and ON-OFF RGCs. D) resting membrane potentials for three RGC types. OFF cell resting membrane potential is significantly lower compared to both ON and ON-OFF cell groups. E) stimulation thresholds for ON and OFF cells. (p*<0.01**, p< 0.001 ***).*

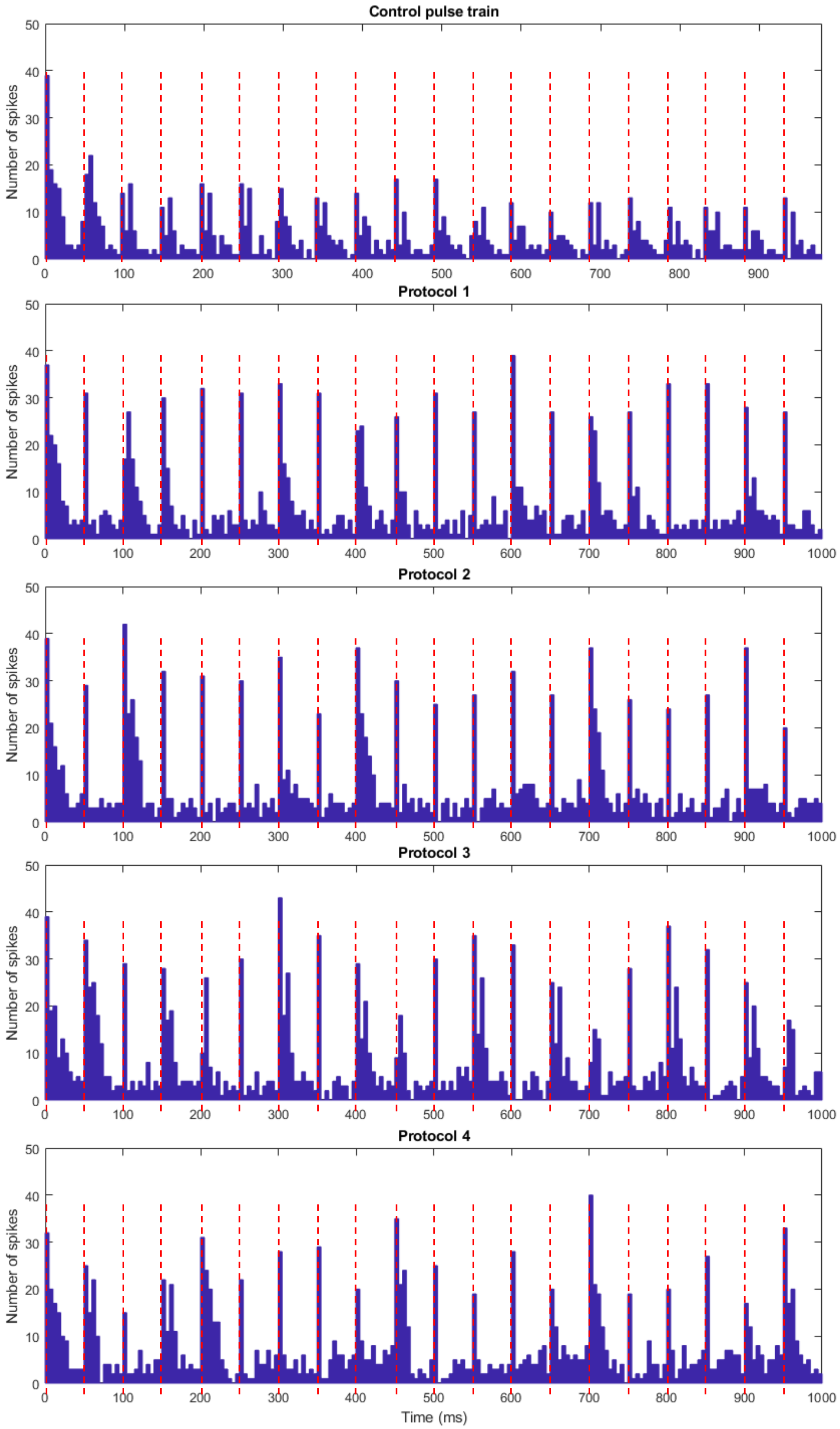

Figure S2. Peristimulus time histogram (PSTH) for the control pulse train and all protocols. The total number of spikes is calculated in 5 ms bins and added across 29 RGCs. Dashed red lines show stimulus times
